# Communicative capital: a key resource for human–machine shared agency and collaborative capacity

**DOI:** 10.1007/s00521-022-07948-1

**Published:** 2022-11-14

**Authors:** Kory W. Mathewson, Adam S. R. Parker, Craig Sherstan, Ann L. Edwards, Richard S. Sutton, Patrick M. Pilarski

**Affiliations:** 1 DeepMind, Montreal, Canada; 2 University of Alberta, Edmonton, Canada; 3 Alberta Machine Intelligence Institute (Amii), Edmonton, Canada; 4 Sony AI, Tokyo, Japan; 5 DeepMind, Edmonton, Canada

**Keywords:** Communicative capital, Machine intelligence, Prostheses, Prosthetic devices, Human–machine interaction, Agency, Prediction learning, Communication, Rehabilitation technology, Robotics

## Abstract

In this work, we present a perspective on the role machine intelligence can play in supporting human abilities. In particular, we consider research in rehabilitation technologies such as prosthetic devices, as this domain requires tight coupling between human and machine. Taking an agent-based view of such devices, we propose that human–machine collaborations have a capacity to perform tasks which is a result of the combined agency of the human and the machine. We introduce *communicative capital* as a resource developed by a human and a machine working together in ongoing interactions. Development of this resource enables the partnership to eventually perform tasks at a capacity greater than either individual could achieve alone. We then examine the benefits and challenges of increasing the agency of prostheses by surveying literature which demonstrates that building communicative resources enables more complex, task-directed interactions. The viewpoint developed in this article extends current thinking on how best to support the functional use of increasingly complex prostheses, and establishes insight toward creating more fruitful interactions between humans and supportive, assistive, and augmentative technologies.

## Introduction

Technology can be used to amplify natural human abilities, provide access to new abilities, and supplement abilities changed due to injury or illness [1–6]. Various tools and technological interventions are well known to support humans in physically interacting with their world, improving perceptual abilities, and supporting decision-making and memory [1, 7–9]. Interventions to provide people with the functions they require for daily life are a core area of interest in rehabilitation, as outlined by the International Classification of Functioning, Disability and Health (ICF) [10, 11]. For example, Geary [1] describes ways that technology is used to enhance sight, touch, hearing, taste, smell, and mental processes. Millán et al. [12], Castellini et al. [13], and Carmena [14] further present views on the use of technology to supplement and enhance motor and sensory abilities for people who have lost body parts or body functions. Of interest to this work are technological advances in assistive or augmentative technology involving tight coupling [15] between a person and a machine with the capacity to learn. This coupling affects the ability of the combined human–machine partnership to have, seek, and achieve goals.

We present the perspective that a human’s ability to have, seek, and achieve goals can be supported using machine intelligence, specifically by combining human ability with reinforcement learning agents [16]. We term this *human–machine shared agency*. This perspective suggests that a human and their machine counterpart should be viewed as partners attempting to accomplish a shared task, where the agency of each partner combines to allow for greater potential capacity to accomplish tasks.

As a main contribution, we introduce *communicative capital: a resource that is built up over time in a human–machine partnership that allows the partners to eventually perform tasks at a capacity greater than either individual could achieve alone*. The resource can consist of accumulated propositional or procedural knowledge, conventions, beliefs, models, and predictions of the other agent. Communicative capital is represented within each agent and is stored within the individual memory of both agents. Communicative capital directly affects the behavioural collaborative capacity of the human–machine partnership.

In this paper, we specifically consider the case where the resource is in the form of predictions learned over time from interaction between human and prosthetic devices. While our setting of interest is human–machine interaction, a helpful motivating example is a human-guide dog partnership that allows both independent agents—human and canine—to accomplish a greater range and complexity of shared tasks (discussed in Sect. 6.1).

## Robotic upper-limb prostheses

**Fig. 1:**
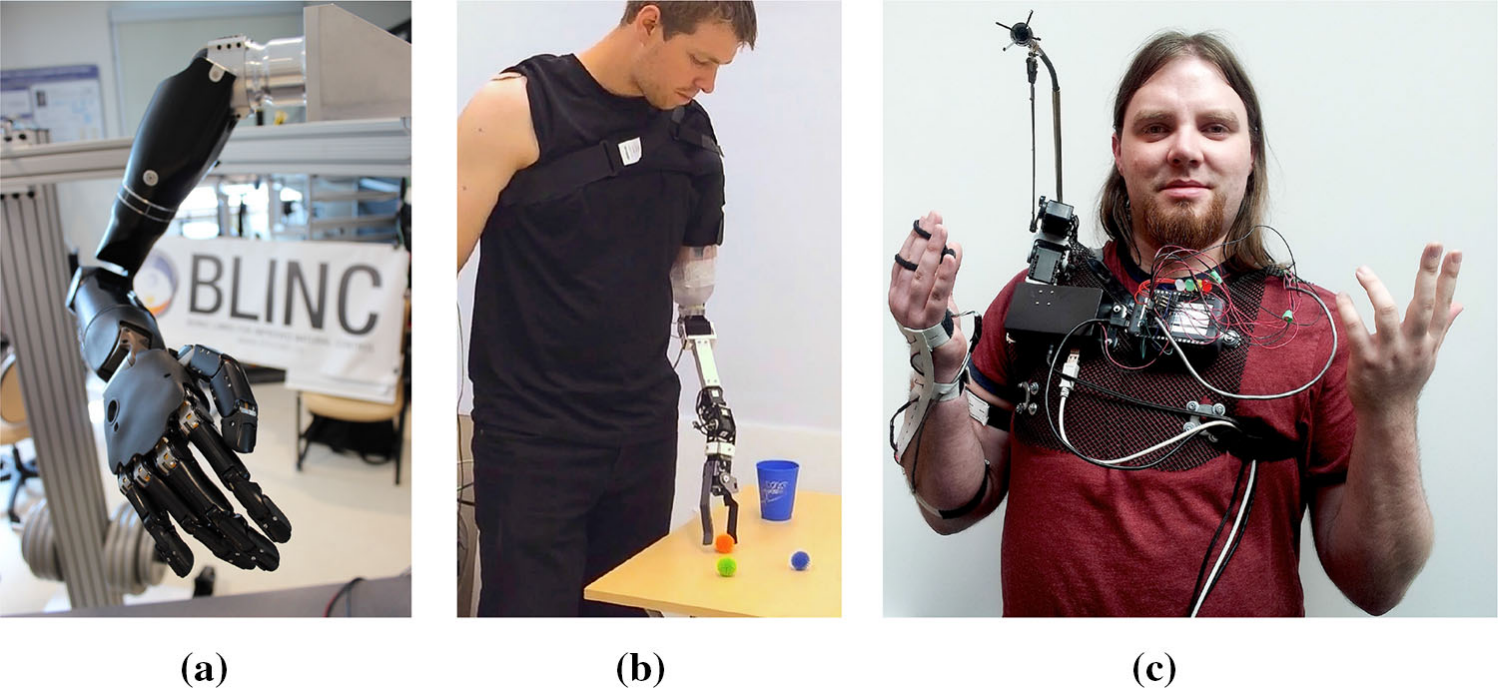
Prostheses examples: **a** Robotic upper-limb prosthetic, **b** Human using a research prosthesis [28], **c** Human using a supernumerary limb [29]

Robotic prostheses and other examples from the field of rehabilitation technology help us focus our thinking on direct human–machine interactions that can be well supported by machine intelligence. The rehabilitation technology setting is appealing in that it involves a direct, immediate, tightly coupled collaboration between a human and their technology to achieve a goal [15, 17]. Examples of assistive rehabilitation devices include semi-autonomous wheelchairs [12, 18], robotic manipulators and locomotors [13, 19], exoskeletons [20], smart living environments [21], and socially assistive robotic coaches [22]. The representative example of assistive rehabilitation technology we focus on in the present work is *robotic upper-limb prostheses*: assistive electromechanical devices attached to the body of individuals with amputations  [23] (Fig. 1). Despite the evolution of prosthetic devices from iron hands to more dexterous mechanical manipulators, and improvements in quality of life for some users, state-of-the-art devices have yet to create a satisfactory solution for many individuals  [13, 24–27].

In the prosthetic setting, movement control contributions from both human and machine must combine effectively in order for the device to benefit the human user. In this setting challenges result from the limited number of degrees of human control and the lack of feedback from the device [13, 30]. The coupling of human and device is further complicated by the dynamic, non-stationary nature of human environments [31]. This coupling has been improved by muscular, neural, and osseointegration allowing for a more direct, high-bandwidth connection between human and machine [13, 19, 27, 32, 33]. To provide a bidirectional flow of information between prostheses and their users, cameras have been used to augment perception [34], microphones and speakers have been used to facilitate natural language interactions [35], and both surgical practices and prosthetic feedback approaches have evolved [30, 36]. Prosthetic devices of the future will receive an unprecedented density of data about human users and their environment, and they should be well equipped to translate such data into actions which support the goals of the users.

Despite the potential of advanced prostheses to support human abilities, current neuroprosthetic literature describes that one remaining limitation on the interaction between human and machine is the number of independent signals flowing between human and machine partners [13]. This constrains control strategy design of upper-limb prostheses to a small number of degrees of freedom, actuated by classification or regression algorithms for real-time control. Giving the upper-limb prostheses some autonomy in their control mechanism has been shown to allow for simultaneous control of multiple degrees of freedom while still using the same number of independent human generated control signals [13]. For example, pattern recognition-based controllers have provided an improvement over conventional controllers in standardized tasks in randomized clinical trials in part because of their ability to learn to interpret and act upon diverse collections of signals provided by a human user [37, 38]. Importantly, these systems therefore require upfront investment on the part of both the device and the user in the form of initial training and subsequent adjustments in order to see the autonomy-related improvements they offer. Increasing the autonomy of a prosthetic device has been shown in many specific cases to significantly increase the capacity of the human-prosthesis partnership to efficiently and effectively accomplish functional tasks [13]. Perhaps surprisingly then, given the diverse data streams and automation capabilities noted above, the specific consequences of prostheses themselves being considered to have and share in agency during human prosthesis interaction has remained relatively under-explored. We now examine the relationship between agency and capabilities in human-prosthesis partnerships.

## Prostheses as agents

In this section, we consider the implications of treating a prosthetic device as an *agent*—an autonomous goal-seeking system. This is not a common perspective—it suggests both sides of a tightly coupled human–machine interface should be thought of as agents with goals. Drawing insight from relationships found in human-human joint action and interaction [39–42], treating a human-prosthesis interaction in this way is in fact not as unfamiliar as it might first seem; with an agent-centric view, each agent would be expected, within its capability, to grow to understand the capabilities of the other and predict how to act accordingly. That is, each agent would naturally and, to the best of its ability, explicitly model the agency of the other to increase the capacity of the partnership in a continual and incrementally increasing fashion. This form of model building and adaptation is present in rather constrained ways in existing state-of-the-art upper-limb prostheses, and something the community hopes to enhance within future prosthetic systems [13].

We first delineate degrees of agency and the resulting capabilities that each side of the prosthetic human–machine partnership may obtain. Here, the human and the machine are considered analogous to co-actors in a joint action task [39–42] or the leader and follower in a two-agent partnership [43]; this collective shared agency is cooperation between a natural and an artificial system [44]. We define agency as the degree to which an autonomous system has the ability to have, seek, and achieve goals. This definition is inspired by the Belmont Report [45], wherein a system assumes agency if it is “capable of deliberation about personal goals and of acting under the direction of such deliberation”. Hallmarks of agency include the ability to take actions, have sensation, persist over time, and improve with respect to a goal. These hallmarks give rise to an agent’s ability to predict, control, and model its environment and other agents. By taking prior perspectives on agency into consideration [46], along with the nuances of the prosthetic setting of interest, we focus on five attributes of agency that may be present in the human or machine agent.

### Be a mechanism

The agent acts in a predetermined way in response to stimulus. For example, a myoelectric controller that processes electromyographic (EMG) signals via a fixed linear proportional mapping to create control commands for prosthetic actuators [47].

### Adapt over time

In addition to being a mechanism, the agent has the capacity to adapt in response to the signals perceived. Through adaptation, the agent may acquire knowledge about its situation (e.g. by modelling and adapting to perceived signals). Adaptation can occur during training, as in the supervised learning of a pattern recognition classifier, or during ongoing experience [13, 48].

### Pursue a goal

The agent has defined goals and an intent to optimize some measure of its own situation. One example of the pursuit of a goal is the maximization of a scalar reward signal, as in computational and biological reinforcement learning [16].

### Model the other agent as adapting

The agent views the other agent as adapting during ongoing interaction. This can alter the way one agent presents signals to the other. For example, a human user trains a pattern recognizing prosthetic with knowledge that the device is adapting to their signals.

### Model the other agent as pursuing a goal

The agent views the other agent as not only changing in response to received signals, but also as pursuing its own objectives. This preliminary theory of mind further alters the way that the one agent presents signals to the other agent.

We present this list of attributes with the caveat that it is likely not exhaustive. We can imagine that there may be higher order attributes of agency which mirror the recursive theory of mind. Additional attributes may parallel high order intentionality and reasoning, as in research in animal ethology, machine theory of mind, and cultural intelligence [49–52]. This line of thinking is discussed further in Sect. 5.3.

**Fig. 2:**
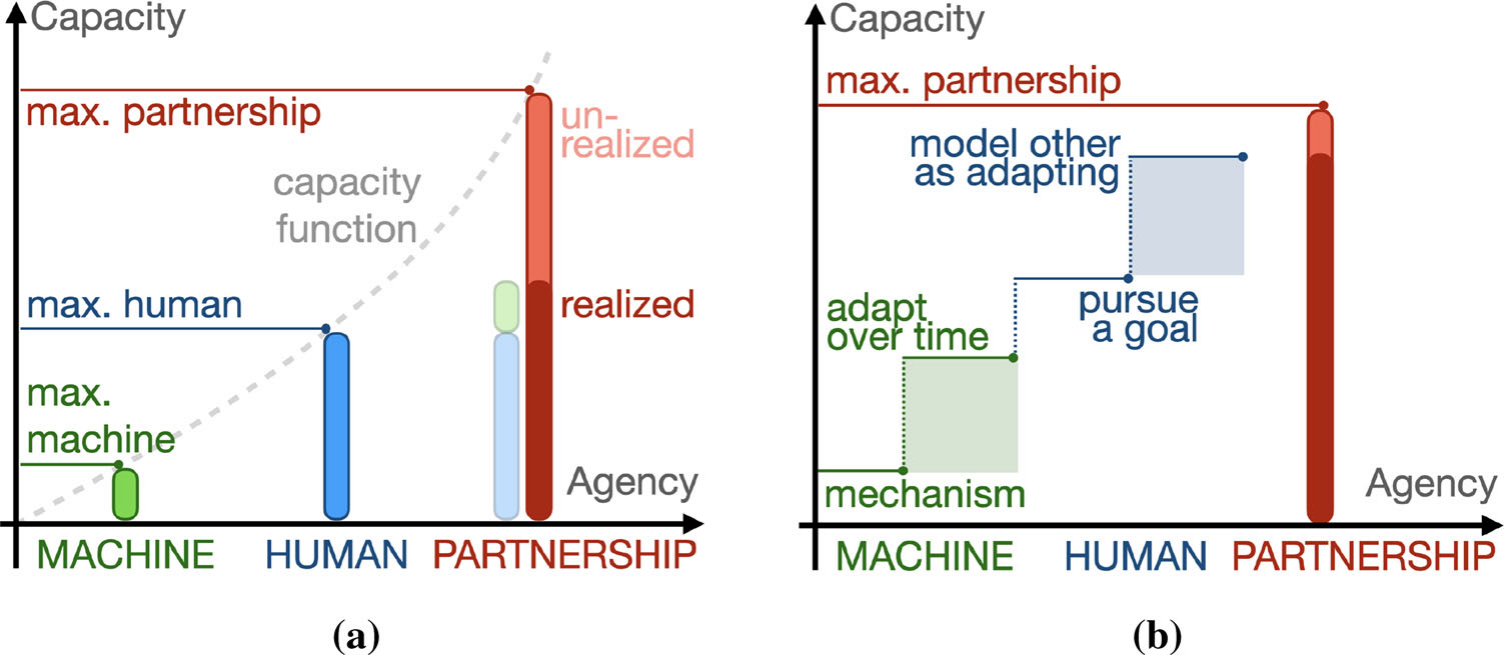
The capacity function (dashed grey line) is the relationship between *capacity* and *agency*. **a** The capacity of the partnership (red) is a function of the contributions from the machine agent (green) and the human agent (blue). **b** Illustrative example of how attributes of human and machine agency can relate to maximum partnership capacity. The light green shaded rectangle represents the capacity increase when a machine agent adapts over time versus when it acts only as a mechanism. The light blue shaded rectangle represents the increase in capacity when a human pursues a goal versus when it also models a machine partner as adapting (Color figure online)

We now outline a schema (Fig. 2) for considering degrees of agency and relate agency to the combined capacity of a human–machine partnership. Capacity and agency in this schema are agnostic to the units of measurement and the exact attributes of agency, so as to be compatible with, and still helpful across, multiple definitions of agency.

*Capacity* is a measure of task performance accomplished by the human–machine partnership as quantified by some metric. *Maximum capacity* is the optimal performance that could be achieved by the partnership, illustrated and labelled ‘max partnership capacity’ in Fig. 2a. This maximum capacity can be realized or unrealized. *Realized capacity* is the actual achieved capacity of the partnership, shown as a solid red bar in Fig. 2a.

### Agency

Agency is the summation of contributions from individual degrees of agency, either discrete or continuous in nature. Multiple degrees combine to increase agency of the agent and shared agency of the partnership (Fig. 2).

### Capacity function

Agency is related to capacity by a *capacity function*. By finding the point on a capacity function corresponding to a given level of agency, we can visualize the maximum capacity of partnership. A system that is a mechanism has less agency and less capacity than a system that is a mechanism, adapts over time, and pursues a goal. A partnership may result in greater capacity than the sum of the two individual systems if both partners model each other and how to effectively utilize the capabilities of both agents. A partnership can also result in a capacity less than the sum of the two individual systems if, for example, the partners interfere with each other.

As an illustrative example, Fig. 2 uses this agency-capacity schema to compare a human-mechanism partnership (without shaded rectangles) to a partnership where the machine is able to adapt (with shaded rectangles). Note how the maximum capacity of the partnership is greater than either could achieve on their own. That capacity may be initially unrealized and change over time, or it might only be realized if both agents can model the other as pursuing a goal.

The way that the goals of the human and machine align is a problem related to team formation in human-human and human-animal partnerships [53]. Such alignment can occur during normal sensorimotor interactions between agents [41, 42, 54, 55]. To examine the process by which such alignment might occur during human–machine interaction, we now introduce the idea of communicative capital. Communicative capital is a resource built up through ongoing interactions between a human and their machine counterpart that correspond to how well both agents understand each other and the partnership [56].

## Communicative capital

**Fig. 3:**
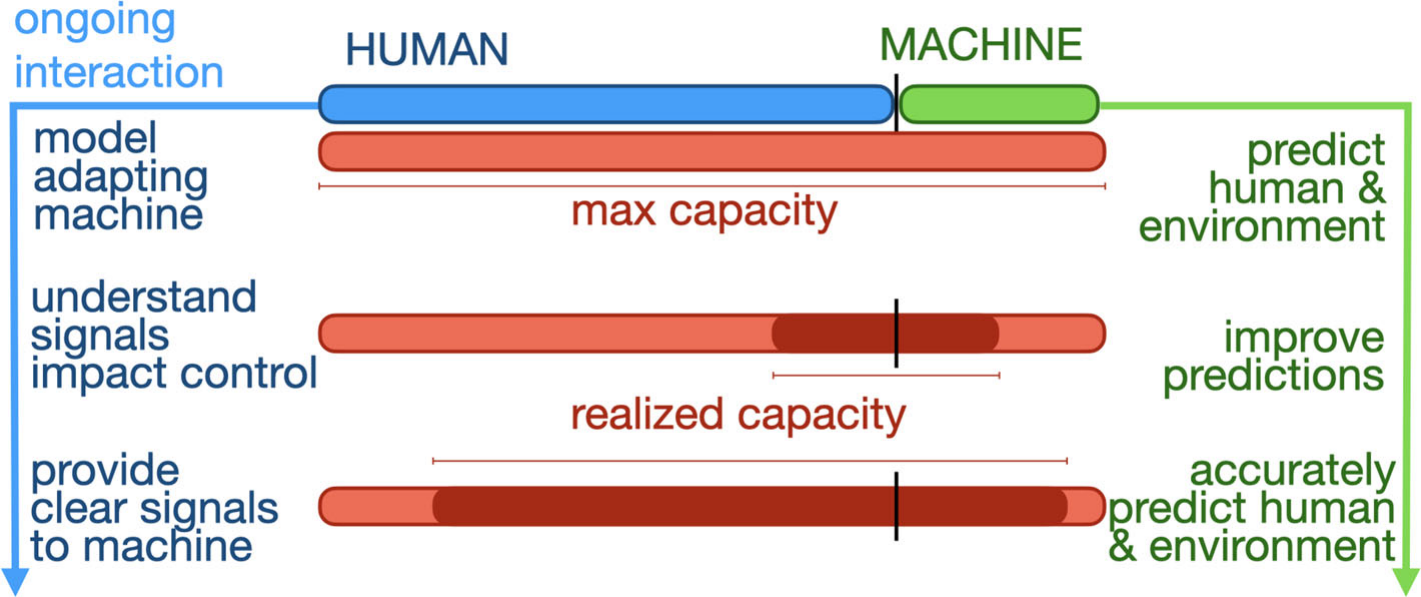
Communicative capital is acquired by the partnership over the course of ongoing interaction. Prior to the partnership interacting, the human (blue) and machine (green) have acquired no communicative capital and thus have no realized capacity. Then, over the course of ongoing interaction (from top to bottom) through modelling, improved predictions, and understanding of the signals of one another, the partnership acquires communicative capital which leads to increased realized capacity (dark red) (Color figure online)

As depicted in Figs. 2 and 3, the agency of the human and the machine contribute to the capacity of the partnership. *Communicative capital* is a resource built through interaction between both sides of the partnership. It enables a partnership to eventually perform a task at a capacity greater than either individual could achieve alone. Accumulating communicative capital requires investment to establish and maintain (see the ‘cost of signalling’ described by Pezzulo and Dindo [41]). The cost of investing in communicative capital may be incurred passively during the interactions of a partnership, or, in many cases, through dedicated effort tangentially related to the ultimate goals of the partnership. For example, users of prosthetic devices learn about the use of their prosthesis before they take it home for use in activities of daily living. In advanced devices that use pattern recognition, teaching both sides of a partnership to engage in a system of meaning-by-convention [57] (e.g., a series of commands to a prosthesis phrased in terms of patterns of myoelectric signals) may require significant additional time and energy but lead to increased future efficiency.

Building communicative capital can also be viewed as a process of compression and decompression, or via the lens of Scott-Phillips et al. [58, 59], one related to ostension and inference. One agent takes an action and thereby encodes information into a signal. The other agent must decode the signal as it arrives, and thereby recover the associated information. To begin to form communicative capital, at least one of the two agents must be able to adapt. Further, we expect the greatest opportunities to build communicative capital will exist when both the human and the machine exhibit the highest possible degrees of agency. We now discuss how communicative capital can be built and used to progressively realize more capacity in prosthetic human–machine partnerships.

## Building capital through interaction

So far we have considered settings where a communication channel exists between the human and the machine. While this channel can be either unidirectional or bidirectional, two-way communication is often beneficial for interactions between multiple goal-seeking agents. If the agent’s goals are not furthered by the information received, then it may ignore the received information. If one agent’s goals are not furthered by what the other agent does with received information, it will choose to not send such information in the future. The agent can send many possible things, and can therefore choose how to balance the cost of sending information with the expected outcomes for itself and the partnership [41]. It follows that both agents should vary their communication to send information that results in both improving with respect to their goals. The variation of communication could be independent, or guided by other parties—e.g., the work of clinical staff to train a patient for prosthesis use, or an instructor helping someone collaborate with a guide dog [60].

In effect, the processes of building communicative capital toward the attainment of goals is about the specification and identification of things each agent cares about, as in “when I do *this* it means *this*”. There can then be a natural progression in the interaction as the two sides get to know each other better. For example, in the progression shown in Fig. 3, improved predictions represent one form of communicative capital. Beneficial collaboration often requires that at least one agent model and predict information about the other. This modelling of the other enables the partnership to achieve tasks with less effort and less explicit communication. This viewpoint is compatible with perspectives on human-human motor coordination [43] and with prosthetic control approaches like pattern recognition [61, 62] as discussed below.

In the following sections, we use the idea of communicative capital and the agency-capacity schema defined in Sect. 3 to examine experimental work where prosthetic control has been improved by ongoing interactions between the device and the user. First, we explore human interactions with *adaptive mechanisms* like pattern recognition systems in commercially available prostheses, and then we detail interactions with *goal-seeking prosthetic agents*.

### Adaptation: prediction enhanced control

First, we consider communicative capital in adaptive control paradigms—specifically, machine learning based prosthetic controllers. There are multiple examples where the human views the machine as adapting and where the machine models and predicts information about the human to better fulfill the human’s intentions [13, 48, 63, 64].

In commercial prostheses with *pattern recognition*, the human engages in a training phase to inform the device about the preferred motions to perform in response to complex patterns of myoelectric activity recorded from the human’s body [13, 62]. The use of pattern recognition can provide users with more intuitive control of their prosthesis [13]. The human becomes more skilled at providing clear training commands, in part because of their knowledge that the machine is learning and adapting from the ongoing interaction. The result is improved capacity due to an increase in communicative capital: the number of human controllable functions can now exceed the number of available degrees of control available in conventional myoelectric control which depends on antagonistic muscle pairs for each degree of freedom [65].

**Fig. 4:**
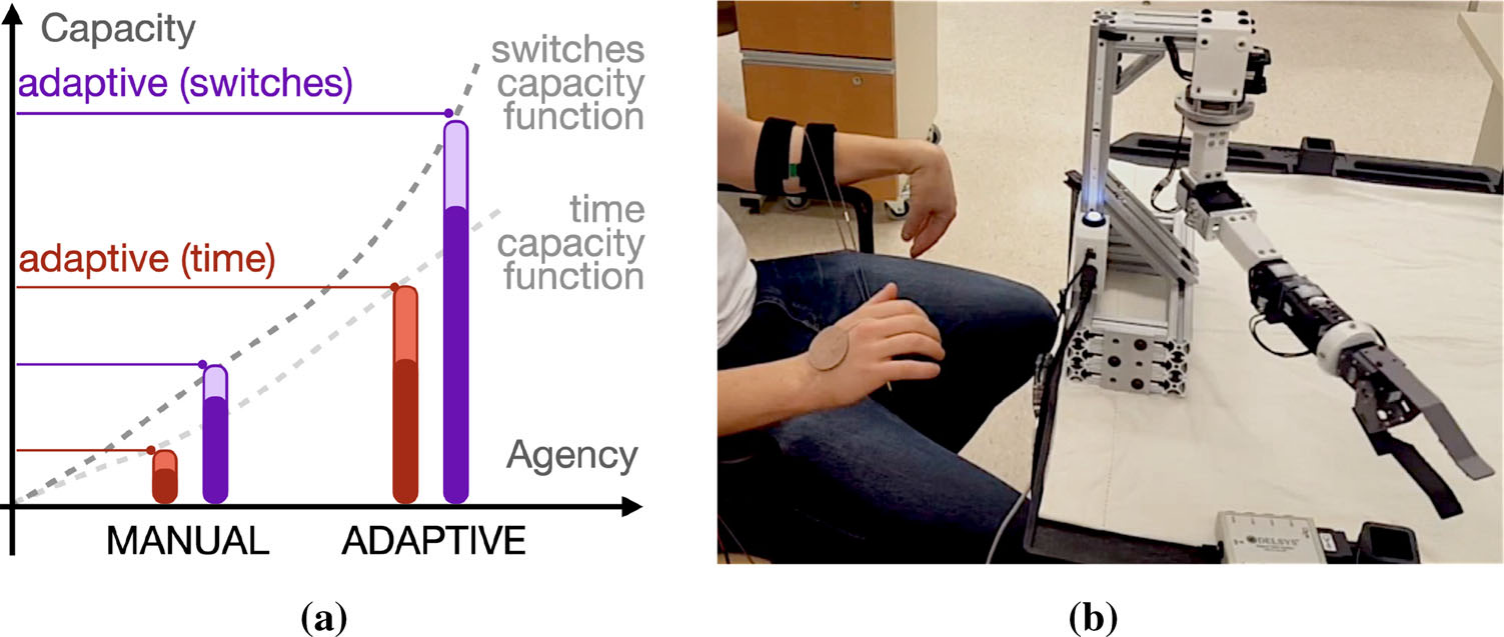
**a** An illustrative example of how adaptive switching enables a prosthetic device to model the way a human uses the functions of a prosthesis and thereby increase agency when compared to the manual switching condition. The increase in shared agency from the manual to adaptive mode of interaction corresponds to increased capacity in terms of (red) time to complete a task, and (purple) the number of switches required to complete a task. This plot shows data approximated from Edwards et al. [66] for illustrative purposes, and (**b**) their participant using the device [63, 64, 66] (Color figure online)

**Fig. 5:**
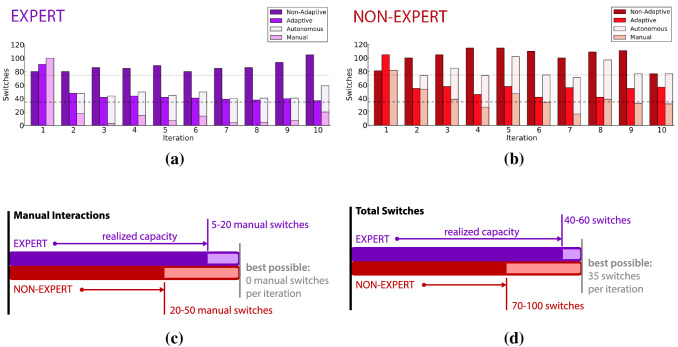
Measured capacity for autonomous and adaptive switching for (**a**) expert and (**b**) non-expert humans (plots adapted from Edwards et al. [66]), summarized in capacity functions relating to (**c**) the number of manual interactions and (**d**) the total number of switches required to complete a control task, expert humans realized more capacity than non-experts

A second example is *adaptive and autonomous switching* [63, 64, 66]. In this setting, a machine learns to make ongoing predictions about how and when a human will decide to switch between controlling one functional joint of a prosthetic device (e.g. the wrist, elbow, or shoulder) and another (Fig. 4). In manual switching, the human uses a separate biophysical control interface to send a ‘change currently controlled joint to the next in a fixed list’ signal to the device. In adaptive switching, the device adapts to the human by suggesting which joint it predicts the user might want to control next. The human’s ability to quickly perform tasks is improved by these suggestions. The device improves its suggestions based on ongoing observations about the human’s actions and preferences. The adaptive nature of the machine, and the increased agency of expert humans to model the machine, lead to increased capacity to successfully complete the task efficiently in terms of reducing both total task time and total switches needed by a human user to complete a task (Fig. 4a,b). In autonomous switching, the device automatically switches which joint is currently controlled. This is done by making and using predictions to automatically switch between the functional control of different prosthetic device joints (see Fig. 5) [63, 66]. Predictions are an acquirable form of communicative capital built up by a machine learning agent during its interactions with a human and the environment.

Observations from both adaptive and autonomous switching suggest that the human begins to model the device as an agent that makes predictions [66]. As human subjects became more familiar, both with their execution of a task and with the role of machine learning as it adapted to a task, they reported greater trust in the autonomy of the device. In these experiments, certain regions of task spaces were observed where the learning system performed with close to 100% prediction accuracy. In these regions, subjects’ behaviour suggested they needed to monitor the prosthetic arm less (e.g., the reduced number of manual switches in Fig. 5).

In the autonomous switching experiments of Edwards et al. [66], users began to predict autonomous switches, often moving the next functional prosthetic actuator prior to hearing a cue alerting them to the machine’s automatic switching behaviour. Increased capacity in terms of reduced manual switching, and the communicative capital that supports it, is evident in users who have extensive prior experience operating adaptive prosthetic devices (see Fig. 5c,d). Users who had a greater understanding of the prosthetic learning system tended to perform actions that benefited learning, allowing the prosthetic arm to build up expectations about their behaviour more swiftly.

Another related example is the work of Sherstan et al. [67]. In this work, a human and a machine learning system share agency in controlling the movement of a robotic arm. The user is only able to control a single joint of the arm at a time and must switch between joints as needed in order to complete a task. The machine agent observes the human’s behaviour and learns to predict the expected joint angles of the robot arm. These predictions are then used to move the arm in collaboration with the human’s own actions [42].

As a final example of adaptive assistive technology related to the upper-limb prosthetic setting, Xu et al. [68] describe a walking-aid robot designed to autonomously adapt to different users. The robot uses reinforcement learning to adjust the relative control of the human in real-time for smoother, faster movement. Smoothness of motion, system safety, and intuitive control can all be viewed as different capacity functions that are improved by the adaptive nature of the machine.

### Goals: reward-based control

Goal-seeking behaviour on the part of both the human and the machine—behaviour driven by processes of reinforcement learning—enables a more detailed progression of interactions than is possible with an adaptive, but not goal-seeking, machine. What follows is one hypothetical progression of the training of an assistive machine, where both the human and the machine are goal-seeking agents, and where the human starts to model the device as a goal-seeking agent. This modelling and adaptation can be observed behaviourally as in the previous section.

1. At the outset, the human can only provide positive feedback (i.e. reward) signals indicating their approval; no other signals have any agreed upon meaning.
2. Using these rewards, the machine can learn a function that maps signals from the human, or other environmental cues, to a valuation that is grounded in cumulative reward (a *value function*, as detailed by Sutton and Barto [16], and used in face valuing by Veeriah et al. [69]).
3. Using this value function, the human teaches the machine a convention that may be used to interact at a low level—e.g., simple commands, body language, cues like pointing, and the basics of shifting between different functions of a system. The human begins to model how their behaviour affects the learning and adaptation of the machine.
4. Using these developed conventions, higher-level abstractions can be established between the human and the machine. These built-up conventions are one component of communicative capital which enable the realization of additional partnership capacity.

With this progression in mind, there are a variety of compatible ways to incorporate human knowledge into a learning system [70–73]. Starting with the idea of training based on primary reward, as in the progression described above, Knox and Stone [74] introduced the *Interactive Shaping Problem*, wherein an agent is acting in an environment and a human is observing the agent’s performance and providing feedback to the agent such that the agent must learn the best possible way to act based on that feedback. The interactive shaping problem is related to communicative capital, as it is a readily observable case of information sharing between two goal-seeking systems with a limited channel of communication.

Goal-seeking behaviour in a machine, and developing communicative capital through the human’s modelling of the machine as goal-seeking agent, increases the maximum capacity of a partnership. A human’s interactions with a machine are supported by a channel of communication with defined semantics (e.g., the reward channel in reinforcement learning [16]) that allows the human to shape the machine’s behaviour in ways that are not possible for an adaptive, non-goal-seeking machine. This communication channel is integral to realizing the goal-seeking agent’s capacity to deal with non-stationary tasks, changing problem domains, and novel environments, in a way that aligns with the human’s goals. Providing the means by which to shape behaviour can also reduce the amount of pretraining for the system, as interactions are now accompanied by online, real-time human feedback. Reward allows the human to shape the machine learning agent to perform the task in a personalized, and situation-specific way—an adaptive goal-seeking agent has the ability to incorporate engineered knowledge, but also move beyond it.

Previous work has demonstrated how both predefined and human-delivered reward could be provided to a goal-seeking agent to gradually improve the control capabilities of a myoelectric control interface [48, 75]. By using a goal-seeking reinforcement learning agent to control the joints of a prosthesis, informed by predictions about future movement, the human–machine partnership was found to be able to progressively refine the simultaneous multi-joint myoelectric control of a robotic arm. In these studies, human approval and disapproval was delivered to the machine with full knowledge of the machine’s learning capabilities. These initial results have been extended to more complex settings which informs how mutual, goal-seeking behaviour supports myoelectric control [76]. These results demonstrate the value of developing communicative capital through the explicit incorporation of human feedback signals. In this representative work communicative capital led to an increased partnership capacity.

### Models, shared agency, and feedback

Beliefs about the nature of internal and external signals are a kind of knowledge that we broadly denote as *models*. Models are required for the higher level attributes of agency; it is useful for a machine to represent, or construct a model, of its partner and the world, in order to achieve more effective interaction. Agent models, as they apply to a human-prosthetic partnerships, may take many forms. They may include, for instance, a collection of learned, temporally extended predictions about the dynamics of the world and the behaviour of the human [16, 77, 78].

As described by Pezzulo and Dindo [54] shared representations may be a critical part of communication during human–machine interaction, and central to the formation of more effective models in terms of beliefs, actions, and intentions. This moves us towards developing a theory of mind—an agent predicting the internal beliefs, motivations, and thoughts of another especially as applied to observable sensorimotor interactions [41, 43, 54]. Recursive theory of mind might imply higher levels of agency, as presented in Sect. 4, and parallel higher order intentionality [49–52]. Future work may explore this other-modelling and how it can be leveraged to build shared knowledge.

As one example of how models can impact a human–machine partnership, Bicho et al. [79] describe a shared construction task in which a robot and a human must work together to assemble a toy. Completion of the assembly task required actions from both agents. The robot infers the goal of the human from contextual clues and acts accordingly, communicating its intention at each point during the task using a speech synthesizer. This allows the human to further model the internal processes of the machine. Another example of a joint task in which a robot infers the goal of the human comes from Liu and Hedrick [80]. In their work, participants and virtual robots collaborate to accomplish a task, and the robot infers the human’s goal based on motion. This research suggests that goal inference (i.e., the modelling goals) decreased the time required to finish tasks and improved other measures of performance, including human–machine trust.

The impact of feedback from an adaptive prosthetic is quantified in work by Parker et al. [81]. In their work, three different kinds of feedback were used to supply a human with information about how best to control the movements of a wearable robot in the form of a supernumerary limb (see Fig. 1c)—no feedback, mechanistic feedback, and adaptive feedback in the form of predictions. The human needed to move the robot in a confined work space, coming as close as possible to the work space’s walls without making physical contact. The human was blindfolded and was acoustically isolated by way of noise-cancelling headphones, so that they only received information about the world via the machine’s feedback.

**Fig. 6:**
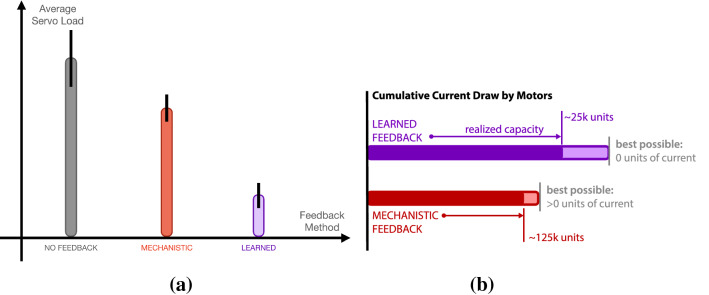
The difference between learned and mechanistic feedback during control of a supernumerary limb (Fig. 1c). **a** Adaptive machine significantly reduced current drawn by the motors of the robot arm (adapted from Parker et al. [81]). **b** Increased machine agency increase realized capacity of the partnership through an investment of communicative capital

The two capacity functions of interest in Parker et al. [81] measured: the current drawn by the motors due to impacts with the work space walls, and the number of times the human was able to use the arm to fully traverse the work space in the given time. On different trials, feedback from the device was either absent, delivered mechanistically upon contact with the walls, or delivered proportional to learned predictions about impacts with the walls. Realized capacity in terms of current draw was found to increase for the case where the human was paired with the adaptive machine, but was found to approach a reduced maximum capacity for the case of mechanistic feedback from the device (see Fig. 6). This work provides insight into how developing communicative capital, specifically through explicitly modelling and increased agency in the delivery of feedback, can influence the maximum capacity possible for a human-prosthetic partnership.

## Discussion

This article has discussed the setting of human–machine interaction, specifically the interactions between a human and their prosthetic technology. However, the ideas presented above regarding agency and communicative capital can be identified and analyzed in the interactions between any two or more intelligent systems. In this section, we provide supporting context from both biological and non-biological examples of how agency plays a role in the interactions of multiple agents to achieve a goal.

### Guide dogs and intelligent assistants

A guide dog could be the oldest documented example of an assistive technology with agency, with an early depiction on the wall of a house excavated in Pompeii dated from c. 79 CE [53, 60]. A guide dog needs to be part of an active partnership—it must have the capability to willingly disobey an instruction when it perceives a danger. The agent in charge of the interaction, human or dog, needs to be able to change from moment-to-moment in order for the partnership to be effective. Because of these desired and atypical behaviours, both the dog and the future owner must be explicitly trained. The human must be taught not only the precise vocabulary understood by the guide dog, but what to expect in response. This requires both parties, human and dog, to invest in communicative capital and learn each others’ idiosyncrasies in order to approach an effective partnership [82].

Computers, whether desktops, tablets, or smartphones, all augment our cognitive abilities. At present, there is significant effort to develop virtual assistants on such devices. Such assistants may have some level of agency; these assistants may be adaptive, changing their behaviour and suggestions to meet the user’s needs [83]. To date, existing computer interfaces have largely remained fixed and unadaptive. However, thanks in part to increases in available computation, computers are now improving in their ability to predict user needs and to provide users with the information and interfaces that are most needed at any given moment [83, 84]. With increased agency, these systems now begin to demonstrate some of the hallmarks of human-human joint action established by the related literature [39, 40, 85].

### Interactive approaches to instruction, communication, and control

There are multiple ways that a human and a machine—e.g., an assistive robot like a prosthesis—can beneficially interact to achieve the human’s objectives [70, 72, 86]. A pertinent family of methods, broadly classified as *interactive machine learning * (IML), has demonstrated the potential to increase the capabilities of decision making systems in complex, dynamic, and novel environments.^1^ In much of the existing IML literature, feedback channels are used as a means by which a non-expert can train, teach, and interact with a system without explicitly programming it. Shaping allows for the human to learn how the system accepts and interprets feedback and for the system to learn the goals of the human [70].

IML has produced a number of important milestones. With respect to goal-driven systems, trial-and-error machine learning has been shown to be accelerated through the presentation of human-delivered reward and forms of intermediate reinforcement. Examples include the use of shaping signals [88], the delivery of reward from both a human and the environment [74], multi-signal reinforcement [70], and combinations of both direct control and reward-based feedback [48, 75, 76]. As described in Sect. 5.2 above, an agent’s learning can be facilitated by a human host through interactive reinforcement learning [74, 89, 90]. Griffith et al. [91] built on the earlier work of Knox and Stone [89] with a framework to maximize the information gained from human feedback. Loftin et al. [92] expanded the space of human interaction through detailed investigation of human teaching strategies and developed systems which model the human feedback. Their systems have been shown to learn faster and with less feedback than other approaches. Interactive learning from demonstrations and instructions have also been shown to help teach different ways of behaving to a learning machine [86, 88, 93–97].

Humans can utilize a number of different approaches to effectively communicate their goals to machine learning agents. Through interactive learning, information from a human can help a machine learner to achieve arbitrary user-centric goals, can improve a system’s learning speed, and can increase the overall performance of a learning system. Advances in IML provide a basis for increasing the rate with which a human-prosthetic partnership may develop communicative capital and thereby realize capacity, and, in certain cases, can also be expected to increase the maximum capacity of a partnership.

### Limitations

There are challenges and limitations in creating machine agents that can build up communicative capital to collaborate more effectively with their human partners. In this section, we highlight several critical areas of focus that should be addressed in future work. Of particular note are challenges related to safely deploying machine learning algorithms in the real-world, especially when deployed on robots tightly coupled to human users. Future work on these algorithms is needed to empirically demonstrate how they are provably robust to a wide variety of environmental factors. As well, mechanisms to align the goals of the human and the machine are critical in shared agency settings. It has been shown in previous research how increasing agency of the machine increases the cognitive demands placed upon the human [76]. Human’s often expect machines to function as mechanisms, unaffected by adaptation. There can be significant implications on their cognitive load once they are required to carry out their own actions as well as model the learning agent [98]. Finally, algorithms deployed in human–machine partnerships will need to adapt quickly to information and signals from the human. Both for reasons of safety, but also because a lack of quick adaptation could lead to human disengagement if the human doesn’t perceive the machine as learning fast enough. Future work on safety, alignment, rapid adaptation, understanding human expectations, and making connections between these systems and modern theories of agency is needed as human–machine partnerships move from the laboratory and into the world. This is true for both prosthetic devices and for collaborative machines more generally.

## Paradigms for evaluation

We expect that increasing the agency of a prosthetic device and investing in communicative capital will allow a collaborative partnership to accomplish tasks faster, easier, more safely, and more efficiently. Work is now needed to test this hypothesis and identify the contributions and practical utility of agency and goal-seeking behaviour on the part of machine learning partner agents. It is our recommendation that researchers design experiments varying the level of agency of both human and the machine in a controlled fashion to assess the contributions from each component of agency. As described in Sect. 5.2, increased agency on the part of the machine enables increased shared agency. This increase is depicted as relative changes in the agency and capacity of both agents.

One means by which to test agent contributions is through the conventional outcome measures used to assess the impact of rehabilitation interventions [99–102]. Outcome measures provide a clearly defined notion of capacity. Further, prosthetic outcome measures are already used to study the benefits of pairing patients to systems with different mechanistic levels of agency (e.g., during prosthetic fitting and patient assessment). In the majority of clinically deployed prostheses, the control approach and system design of the device is fixed. The communicative capital of the mechanism—how it interprets body signals and maps them to actuators—provides immediate realized capacity at a level determined by the mechanism’s designers. Measures like the Southampton Hand Assessment Procedure, the Box-and-Blocks Task, and others are used to provide a quantitative assessment of the impact of these prosthetic mechanisms [102, 103]. Recent developments in the assessment of gaze and movement have further shown concrete, capacity-related metrics that evaluate user-prosthesis abilities via changes in the relationship between biomechanics and visual attention, as well as other measurable correlates of perceived control and agency [104–107]. Some of these measures have been shown to serve as proxies for the state of human predictive models of their machine partner, and thus may provide a way to quantify communicative capital as it is built by the human side of a human–machine partnership [104]. Rigorous, incremental testing of agency is therefore highly compatible with existing approaches, and will be significantly extended as more comprehensive motor, sensory, and cognitive outcome measures are developed.

One fruitful avenue for experimentation, as explored in Parker et al. [29], is to deliberately reduce the agency of the human by removing control options and/or sensory inputs as they complete a task. In this way, the authors were able to elucidate how different levels of agency in the machine contribute to the performance of the partnership. A second, complementary paradigm is to dramatically increase the agency of the machine beyond what is technically possible, so as to study the outcomes and conditions that support shared agency. One way to do this is a type of sham trial known as a *Wizard-of-Oz* experiment (e.g. Viswanathan et al. [18]). Paradigms for evaluating human–machine partnerships will continue to develop as technology supporting shared agency evolves. We now conclude with several brief reflections.

## Conclusions

We argue that tightly coupled human–machine partnerships, such as humans and prostheses, should be thought of as adaptive multi-agent systems where the agency of human and machine combine to achieve more capacity than either could independently. We present an agency-capacity schema that relates shared agency to the capacity of human–machine partnerships, and we show how communicative capital is the key resource that a partnership needs to invest in to access the full capacity of the combined agency of the pairing. Using examples from the literature, we illustrate how increases in the agency of a prosthesis can tangibly improve the capabilities of its human user. We highlight three main conclusions from this work as novel contributions supporting human-prosthesis interaction: (1) we propose that designing assistive devices as goal-seeking agents improves the range of possibilities for robust and flexible interaction, (2) we argue that an agent-based viewpoint of human–machine interaction enables a structured progression toward more capable partnerships between people and devices, and (3) we describe how communicative capital is a resource built through ongoing human–machine interaction which enables a partnership to eventually perform tasks at a capacity greater than either could individually. Machine intelligence enables the acquisition and use of communicative capital in human-prosthesis partnerships to more effectively and more efficiently accomplish tasks. We believe the agency-based viewpoint on assistive technology proposed in this work contributes unique and complementary ideas to the development of highly functional human–machine partnerships. Designers and developers should construct systems which actively invest in communicative capital as such investment will lead to increases in shared agency to achieve more capacity than they would be able to otherwise.

## Data Availability

Not applicable.

## Footnotes

1 Though, some argue that **all machine learning is interactive machine learning** because humans interact with machines through every step of the design, development, deployment, and dissemination of such systems [87].
